# Application of the Environmental Relative Moldiness Index in Finland

**DOI:** 10.1128/AEM.02785-15

**Published:** 2016-01-07

**Authors:** Martin Täubel, Anne M. Karvonen, Tiina Reponen, Anne Hyvärinen, Stephen Vesper, Juha Pekkanen

**Affiliations:** a Living Environment and Health Unit, National Institute for Health and Welfare, Kuopio, Finland; b Department of Environmental Health, University of Cincinnati, Cincinnati, Ohio, USA; c Department of Environmental Science, University of Eastern Finland, Kuopio, Finland; d United States Environmental Protection Agency, National Exposure Research Laboratory, Cincinnati, Ohio, USA; e Department of Public Health, University of Helsinki, Helsinki, Finland

## Abstract

The environmental relative moldiness index (ERMI) metric was previously developed to quantify mold contamination in U.S. homes. This study determined the applicability of the ERMI for quantifying mold and moisture damage in Finnish residences. Homes of the LUKAS2 birth cohort in Finland were visually inspected for moisture damage and mold, and vacuumed floor dust samples were collected. An ERMI analysis including 36 mold-specific quantitative PCR assays was performed on the dust samples (*n* = 144), and the ERMI metric was analyzed against inspection-based observations of moisture damage and mold. Our results show that the ERMI was significantly associated with certain observations of visible mold in Finnish homes but not with moisture damage. Several mold species occurred more frequently and at higher levels in Finnish than in U.S. homes. Modification of the ERMI toward Finnish conditions, using a subsample of LUKAS2 homes with and without moisture damage, resulted in a simplified metric based on 10 mold species. The Finnish ERMI (FERMI) performed substantially better in quantifying moisture and mold damage in Finnish homes, showing significant associations with various observations of visible mold, strongest when the damage was located in the child's main living area, as well as with mold odor and moisture damage. As shown in Finland, the ERMI as such is not equally well usable in different climates and geographic regions but may be remodeled to account for local outdoor and indoor fungal conditions as well as for moisture damage characteristics in a given country.

## INTRODUCTION

Moisture problems in Finnish homes are common. A study relying on standardized building inspections reported signs of current or previous moisture fault in 80% of residences, with >50% of these homes being in need of repair (1). A more recent analysis from a Finnish cohort confirmed that >70% of homes would be in need of repair beyond just esthetic interventions, and mold was visually observed in 38% of these homes (2). Moisture problems appear to be prevalent also in Finnish schools (3). This implies a need to prioritize renovation actions based on the severity or extent of the moisture problem and/or the related health hazard. Exposure to water-damaged, moldy buildings has been linked to both exacerbations and development of asthma (4–8). Identifying and quantifying “abnormal” mold exposures might be critical in efforts to reduce the disease burden of asthma due to dampness and moisture damage in buildings.

Many techniques have been used to estimate mold contamination in homes, but self-reporting of visual observations is the most commonly used method. A visual assessment, often supported by microbial verification of damage situations, can be accurate if performed by an experienced building inspector or engineer and in such cases can be considered the current gold standard for assessing indoor mold. However, not everyone performing visual inspections is equally qualified, and mold contamination can be hidden from sight inside structures (9). Moreover, in large epidemiological studies, detailed building inspections are typically not feasible.

Currently, cultivation-based detection of viable fungi and bacteria from indoor samples such as building material or air samples is the most commonly used method for quantifying microbes in support of building investigations. The cultivation-based approach has drawbacks, however, including extended analysis durations, limited reproducibility (especially considering short-term air samples), and the fact that only the alive and cultivable fractions of the microbial spectrum are visualized. In conclusion, there is a need for improved and objective metrics for quantifying mold contamination in homes.

The U.S. Environmental Protection Agency (EPA) together with the U.S. Department of Housing and Urban Development established a metric to quantify mold contamination in U.S. homes, called the environmental relative moldiness index (ERMI) (10). For ERMI analysis, a DNA-based technology, mold-specific quantitative PCR (qPCR) (MSQPCR), is used to measure the concentrations of 36 indicator molds in floor dust samples. Of the 36 molds, 26 are group 1 species commonly found at higher levels in water-damaged homes and 10 are group 2 species commonly found in U.S. homes, independent of water damage (11).

The ERMI metric has been used in many studies in the United States as a predictor of moisture damage, mold contamination, and asthma (12–15). The ERMI has also been applied in a few studies outside the United States (16–18). However, the categorization of the ERMI mold species and groups into water damage (group 1) and normal background (group 2) molds was developed in a restricted sample of moisture-damaged and reference homes in a confined geographical area in the United States (Cleveland, OH) (11). Thus, the applicability of the ERMI metric in different countries or regions with differences in climatic conditions, building stocks, and characteristics of moisture damage and mold contamination needs to be explored. The purpose of this analysis of homes of a Finnish birth cohort (19) was to determine if the ERMI metric might be applied to quantifying moisture damage and mold contamination in Finnish homes.

## MATERIALS AND METHODS

### Study population.

The LUKAS2 study is an ongoing birth cohort study in Eastern Finland, with the mothers of the study subjects recruited at Kuopio University hospital (20). The mothers were monitored from the third trimester of pregnancy, and children were born between May 2004 and May 2005. Written informed consent was obtained from the parents; all study protocols for the LUKAS2 study were approved by a local ethics committee in Finland (20). The cohort consists of a general population sample of homes in rural and suburban areas in this region, excluding high-rise apartment buildings. A total of 199 dust samples were collected during early childhood; 144 of these samples were included in this analysis, based on following criteria: sufficient dust was available for DNA extraction and qPCR analyses, corresponding results from home inspection were available, the families had lived in the same home at the time of home inspection and dust vacuuming, and the homes were nonfarming homes.

### Home inspection for dampness and mold.

During early childhood (mean child age, 9 months), a building engineer performed detailed “walkthrough” inspections of the study homes to assess moisture damage, visible mold, and other dampness indicators, as previously described in detail (2, 19). The inspections were performed according to a standardized protocol (1) and by utilizing standardized checklists and questionnaires (5). In brief, visual observations in individual rooms and areas of the home (bedroom, living room, kitchen, and bathroom, etc.) were complemented by recording of surface moisture, visible mold, and mold odor. Moisture damage observations were graded based on extent and severity. The inspector recorded detailed estimates of individual damage in each location separately and also made an overall assessment of the whole house. A 6-point “need-for-repair” scale (1) was used to grade the severity of moisture damage both for individual observations and, on a more general basis, for the house as a whole: classes 0 and 1 refer to damage with no need for repair or only cosmetic repair, class 2 means that repair of surface materials is needed, class 3 indicates that repair of structural components is needed, and classes 4 and 5 call for more extensive repairs due to moisture problems.

“Moisture damage” was categorized into three levels (none, minor, or major) by combining the 6-point need-for-repair estimation scale (1) and the area of damage (5). “No damage” was defined as need-for-repair class 0 or 1. “Major damage” was defined as need-for-repair class 2 and an area of damage of ≥1 m^2^, need-for-repair class 3 with an area of damage of ≥0.1 m^2^, or need-for-repair class 4 or 5. Damage other than those described above was classified as “minor damage.” In the case of several damage observations in a given location, the areas of damage with same need for repair were summed up. “Visible mold” was categorized as “yes” (observation of moisture damage with mold spots only or with more extensive visible mold) or “no” (no observed moisture damage with visible mold). Mold on silicone sealants in the kitchen or in the bathroom only was classified as no mold. “Mold odor” was categorized as “no odor,” “slight odor,” or “odor.” Combination variables of observations made in the “child's main living areas” were created, combining moisture damage or visible mold observations for the child's bedroom, living room, and kitchen.

### Dust sample collection and MSQPCR analysis.

The protocol for dust collection was described previously (20). Parents took samples from living room floors of homes of the children when the children were ∼1 year old. The dust sample was vacuumed from a 1-m^2^ area of a rug for 2 min (in cases where there was no rug in the living room, the sample was taken from a 4-m^2^ area of smooth floors for 2 min), using a regular vacuum cleaner and polyester dust sampling socks (Allied Filter Fabrics Pty. Ltd., Australia). The dust samples were processed at the National Institute for Health and Welfare in Finland, where they were stored in the dark at 4°C prior to sieving and then kept in a desiccator for 2 days. The dust samples were then stored frozen at −20°C until shipment on dry ice to the U.S. EPA.

Each dust sample was sieved through a 300-μm-pore-size nylon mesh (Gilson Company, Inc., Lewis Center, OH), and 5.0 ± 0.1 mg of sieved dust was then extracted and DNA was purified by using the DNA-EZ extraction kit (GeneRite, Monmouth Junction, NJ), according to the manufacturer's instructions. The concentration of each mold was determined by MSQPCR analysis (21). The standard reaction assay mixtures contained 12.5 μl of Universal master mix (Applied Biosystems, Inc., Foster City, CA), 1 μl of a mixture of forward and reverse primers at 25 μM each, 2.5 μl of 400 nM TaqMan probe (Applied Biosystems, Inc.), 2.5 μl of 2 mg/ml of fraction V bovine serum albumin (Sigma Chemical, St. Louis, MO), and 2.5 μl of DNA-free water (Cepheid, Sunnyvale, CA). Five microliters of the DNA extract from the sample was added to this mix. All primer and probe sequences used in the assays were reported previously (22). Primers and probes were synthesized commercially (Applied Biosystems, Inc.).

The ERMI value for each home was calculated by taking the sum of the logs of the concentrations of the 26 group 1 species (*s*_1_) and subtracting the sum of the logs of the concentrations of 10 group 2 species (*s*_2_) (10):

(1) $$\text{ERMI}\;=\;\overset{26}{\underset{i\;=\;1}{\Sigma }}\log_{10}(S_{1i})\;-\;\overset{10}{\underset{j\;=\;1}{\Sigma }}\log_{10}\left(S_{2j}\right)$$

### Comparison of ERMI molds in Finland and the United States.

The occurrence (percentage of samples in which the mold was detected) and population geometric mean (GM) for each of the 36 ERMI molds in these homes in Finland were compared to the occurrence and population GM for these same molds in homes in the United States, using data that were previously reported (10).

### Development of the FERMI.

In order to adapt the ERMI metric and improve its applicability to local conditions in Finland, we selected a subsample of LUKAS2 homes with severe moisture damage (i.e., moisture-damaged homes [MDHs]) (*n* = 20) and reference homes (RHs) (*n* = 42) that had no signs of moisture damage in any room of the house. Severe moisture damage was defined as major moisture damage, visible mold, and/or mold odor in the main living areas (i.e., kitchen, living rooms, bedrooms, and main hallways connecting these rooms). As in the definitions of the original ERMI, we (i) included only mold species (i.e., qPCR assays) with a mean value in these 62 homes of ≥1 conidium per 5 mg of dust (based on this criterion, Aspergillus unguis was excluded from further analyses) and (ii) calculated geometric mean ratios for moisture-damaged versus reference homes for 35 individual mold species to define group 1 (moisture damage-associated) and group 2 (background) mold species.

### Statistical analysis.

Geometric means were calculated from log-transformed qPCR results; zero values in qPCR data were also recorded as zero values in the log-transformed data set. ERMI and FERMI values were normally distributed, and thus, a *t* test or one-way analysis of variance (ANOVA) was used to compare mean values for moisture damage indicators in the sample of 144 LUKAS2 study homes. The analyses were performed with SAS version 9.3 (SAS Institute, Cary, NC).

## RESULTS AND DISCUSSION

In this analysis, we show a basic agreement of the environmental relative moldiness index (ERMI) with inspection-confirmed, visible mold observations in Finnish homes but not with moisture damage. We demonstrate a substantial improvement of this metric by adapting it to local conditions by redefining what can be considered moisture damage and background molds based on sets of severely moisture-damaged homes and reference homes in Finland.

The ERMI has been developed to quantify mold contamination in homes in the United States (10). It is a metric that is based on quantitatively measured mold species or groups that are either linked to conditions of moisture and mold damage or considered normal background molds, with these definitions being based on a sample of U.S. homes (11). By integrating a large number of different mold taxa potentially linked to moisture damage, the ERMI metric takes into account the complexity of microbial exposure situations and acknowledges the fact that no two moisture-damaged homes are identical in terms of their extent and profile of microbial contamination (23). This approach also factors in the content of mold species that are commonly observed in homes independent of moisture or mold damage and by doing so adjusts for high fungal levels in homes that are not linked to moisture problems but rather are related to outdoor or other sources.

The ERMI metric has been applied to many studies of mold contamination and also of occupant asthma in U.S. homes (12–15). While the ERMI has not been tested or reported extensively in countries outside the United States, the metric has been shown to be useful in studies of mold contamination in some European countries, including the United Kingdom, specifically Scotland (18), and France (16). This study is the first to explore the applicability of the ERMI in Finland, in a Northern climate.

The mean ERMI values were generally higher in Finnish homes where visible mold or moisture damage was observed than in homes without such observations. However, the ERMI was statistically significantly higher (*P* = 0.007) only if there was visible mold in the child's main living areas, but other associations, especially with minor or major moisture damage, did not reach statistical significance (Table 1). These findings indicate that while the ERMI metric responds to increases or changes in microbial content due to moisture problems in a Finnish home, a clear numerical response in this assessment can be observed only in very severe cases that manifest as visible mold growth. Floor dust samples for ERMI determination were collected in the living room; the definition of the “child's main living area” includes the living room, the area next to the child's bedroom, and the kitchen area in the home. Thus, it is conceivable that visible mold observed in the child's main living area correlates strongly with the ERMI metric. The fact that visible mold observed in the living room, however, failed to statistically significantly increase the living room floor dust ERMI can be explained by the low number of observations of visible mold in living rooms (*n* = 3) in this study, heavily limiting the statistical power in calculating these associations.

**TABLE 1:** Comparison of mean ERMI values in Finnish LUKAS2 homes categorized based on observations of visible mold or moisture damage in the living room, the child's main living areas, and the whole house^*a*^

Area of home	Visible mold and ERMI				Moisture damage and ERMI			
	Detection	No. of homes	ERMI^*b*^	*P* value	Detection	No. of homes	ERMI^*b*^	*P* value
Living room	No	141	5.43		None	128	5.27	
	Yes	3	10.24	0.52	Minor	12	7.45	
					Major	4	8.22	0.21
Child's main living area	No	134	5.22		None	102	5.35	
	Yes	10	9.72	0.007	Minor	31	5.22	
					Major	11	8.12	0.22
Whole house	No	100	5.14		Class 0/1	60	5.16	
	Yes	44	6.42	0.17	Class 2	50	5.72	
					Class ≥3	34	5.90	0.76

a Differences in the mean ERMI values were evaluated by using a *t* test or one-way ANOVA.

b Mean ERMI value.

We observed considerable differences in the profiles of mold species, analyzed in the same laboratory, by comparing the results from the Finnish LUKAS2 cohort to the results of a large national survey in the United States (10). The occurrences and population geometric means for each of the 36 ERMI molds for the Finnish homes compared to those for 1,096 U.S. homes are shown in Table 2. These differences in prevalence and levels of different mold species that constitute the ERMI likely contribute to why a mold scale developed in the United States does not predict indoor mold conditions in Finland equally well. Molds with GM levels at least 10 times higher in Finnish homes than in U.S. homes included four ERMI group 1 (moisture damage) species, Aspergillus restrictus, Aureobasidium pullulans, Penicillium brevicompactum, and Trichoderma viride, and two ERMI group 2 (background) species, Cladosporium cladosporioides (Type 1) and Cladosporium herbarum. Aspergillus penicillioides had a GM level that was 10 times higher in the United States than in Finland (Table 2). Qualitative and quantitative differences in outdoor and indoor microbes in different geographic areas and/or climates are well-known phenomena (24–27). Building characteristics, including, for example, predominant construction types, building materials, or ventilation strategies, and building use vary between climates, countries, and cultures. As a consequence, the types of moisture problems and their manifestations and associated microbial growth may also vary, depending on the country/region/climate in which the building is located (3, 28).

**TABLE 2:** Comparison of the occurrences and geometric mean cell equivalents per milligram for the 36 ERMI molds in LUKAS2 homes in Finland (*n* = 144) compared to U.S. homes (*n* = 1,096)^*a*^

Mold	% occurrence		GM concn (cell equivalents/mg)	
	Finland	U.S.	Finland	U.S.
Group 1				
Aspergillus flavus	44	36	1	2
Aspergillus fumigatus	44	62	2	3
Aspergillus niger	67	69	4	4
Aspergillus ochraceus	38	27	3	2
Aspergillus penicillioides	95	90	4	91
Aspergillus restrictus	95	12	1,109	2
Aspergillus sclerotiorum	5	26	1	2
Aspergillus sydowii	15	29	1	3
Aspergillus unguis	6	20	1	2
Aspergillus versicolor	13	30	1	2
Aureobasidium pullulans	100	94	3,485	263
Chaetomium globosum	44	51	2	2
Cladosporium sphaerospermum	88	82	5	13
Eurotium amstelodami	100	98	21	155
Paecilomyces variotii	31	46	1	2
Penicillium brevicompactum	98	52	276	5
Penicillium corylophilum	50	17	2	2
Penicillium crustosum group	43	8	5	1
Penicillium purpurogenum	19	15	1	1
Penicillium spinulosum	22	20	1	1
Penicillium variabile	31	50	1	3
Scopulariopsis brevicaulis	66	53	1	2
Scopulariopsis chartarum	52	38	2	2
Stachybotrys chartarum	24	35	1	2
Trichoderma viride	95	27	86	2
Wallemia sebi	98	75	176	18
Group 2				
Acremonium strictum	82	57	3	4
Alternaria alternata	96	88	18	35
Aspergillus ustus	28	40	2	2
Cladosporium cladosporioides type 1	100	99	4,540	331
Cladosporium cladosporioides type 2	100	70	24	4
Cladosporium herbarum	100	84	2,419	31
Epicoccum nigrum	100	93	68	117
Mucor group	99	92	37	15
Penicillium chrysogenum	38	66	2	5
Rhizopus stolonifer	53	29	2	1

a See reference 10.

One key aspect in official guidance that regulates and provides advice on building investigations conducted in the context of moisture damage and indoor mold contamination in Finland is that whenever mold is visibly observed, no further microbial confirmation is necessary or required. In such cases, mold-contaminated material is requested to be cleaned or removed, and the source of the moisture and mold problem needs to be corrected. Following this philosophy, there is little need for microbial assessment tools that would confirm observations of visible mold. On the other hand, given that a large percentage of the Finnish building stock is affected by some level of moisture damage (1, 2), often accompanied by mold growth that is hidden in structures, there is a large demand for approaches that would allow detection of the occurrence of a potentially abnormal microbial source in a building and facilitate an objective grading of moisture and mold damages of various degrees. This could, for example, allow prioritization of renovation measures.

Considering the only moderate performance of the original ERMI scale with respect to moisture damage and taking into account the obvious differences in fungal occurrence between the United States and Finland, we aimed to redefine the ERMI metric for local Finnish conditions. We utilized a subset from the LUKAS2 study composed of homes with severe moisture damage in main living areas (*n* = 20) and non-moisture-damaged reference homes (*n* = 42). In the original ERMI definitions, mold species with a geometric mean ratio of >1 in moisture-damaged versus non-moisture-damaged homes were categorized as group 1 (moisture damage) molds, and those with a GM ratio of <1 were categorized as group 2 (background) molds (10). When these definitions were applied to Finnish homes with and without moisture damage, it was striking to observe that 11 out of 25 mold species that were associated with moisture damage in the United States (group 1 molds) would be classified as background molds (group 2) in Finland (Table 3). Similarly, several of the original ERMI group 2 molds were shown to respond to moisture damage conditions in Finnish homes (switch from group 2 to group 1). Moreover, we observed that the grouping of mold species into either group 1 or group 2 was not consistent for some of the molds but varied depending on the season (snow cover or not) when the floor dust sample was collected for mold analysis (Table 3). This is probably due to the large differences in Finnish climatic conditions between seasons, where a permanent snow cover for several months largely reduces the influx of microbes from outdoors into homes (29). Based on these observations, our approach toward creating a Finnish ERMI included (i) reallocating the mold species into moisture damage and background molds based on Finnish conditions using the above-mentioned sample of Finnish homes with clear moisture damage and without any moisture observations; (ii) including only molds with a clear association with moisture and mold damage in group 1 (mold species with a GM ratio of damaged to nondamaged homes of >1.5 were considered good moisture damage indicators and were allocated into group 1), with this association being independent of the season of floor dust sampling (exclusion of mold species that provided obviously opposing estimates in the GM ratios in winter and nonwinter samples from further analyses [i.e., GM ratio clearly >1 during snow cover and clearly <1 during non-snow cover or vice versa]); and (iii) including only such mold species as background molds in group 2 that showed no association with moisture damage (GM ratio of ≤1) independent of season and that were well prevalent (>50%) in the house dust samples (Table 3).

**TABLE 3:** Original ERMI mold species (group 1 and group 2) measured in floor dust from a sample of Finnish homes in the LUKAS2 cohort with severe moisture damage (MDHs) and nondamaged reference homes (RHs)^*a*^

Mold	Prevalence (% samples >DL)^*b*^	GM concn (no. of conidia/5 mg)		GM ratio of MDHs/RHs		
		Moisture-damaged homes (*n* = 20)	Reference homes (*n* = 42)	All samples (all seasons) (20/42)	Samples taken during snow cover (4/13)	Samples taken during no snow cover (16/29)
ERMI group 1						
Aspergillus flavus	47	1.48	1.53	0.96	0.85	0.99
Aspergillus fumigatus	50	2.06	2.38	0.87	1.09	0.80
Aspergillus niger	77	5.65	5.45	1.04	1.32	0.92
Aspergillus ochraceus	39	6.27	2.42	2.59	3.30	2.53
Aspergillus penicillioides	97	3.90	4.39	0.89	2.02	0.67
Aspergillus restrictus	97	1,503.68	1,171.24	1.28	6.80	0.99
Aspergillus sclerotiorum	6	1.04	1.12	0.92	0.83	0.96
Aspergillus sydowii	16	1.26	1.20	1.06	0.81	1.14
Aspergillus versicolor	19	2.70	1.30	2.08	1.48	2.18
Aureobasidium pullulans	100	4,355.01	4,562.12	0.95	3.16	0.57
Chaetomium globosum	47	2.35	1.57	1.50	0.95	1.77
Cladosporium sphaerospermum	85	10.93	4.55	2.40	18.40	1.43
Eurotium amstelodami	95	22.32	21.63	1.03	4.00	0.83
Paecilomyces variotii	37	1.34	1.53	0.88	1.14	0.79
Penicillium brevicompactum	98	169.09	323.15	0.52	1.31	0.48
Penicillium corylophilum	50	3.59	1.87	1.92	1.78	1.93
Penicillium crustosum group	50	8.51	5.09	1.67	2.16	1.70
Penicillium purpurogenum	26	1.13	1.35	0.84	0.63	0.93
Penicillium spinulosum	31	0.84	0.96	0.88	0.89	0.87
Penicillium variabile	31	1.20	1.25	0.95	0.90	0.95
Scopulariopsis brevicaulis	71	0.86	0.91	0.94	1.81	0.76
Scopulariopsis chartarum	63	3.49	3.25	1.08	2.79	0.79
Stachybotrys chartarum	24	1.73	1.24	1.40	2.45	1.16
Trichoderma viride	97	114.74	113.91	1.01	1.16	1.18
Wallemia sebi	100	209.22	203.15	1.03	9.77	0.55
ERMI group 2						
Acremonium strictum	87	4.06	3.85	1.05	2.89	0.78
Alternaria alternata	95	15.89	17.72	0.90	0.71	0.91
Aspergillus ustus	27	1.54	1.58	0.97	1.46	0.87
Cladosporium cladosporioides 1	100	3,506.15	6,355.21	0.55	0.99	0.40
Cladosporium cladosporioides 2	100	26.10	19.14	1.36	1.16	1.42
Cladosporium herbarum	100	1,705.70	2,924.37	0.58	1.23	0.41
Epicoccum nigrum	100	43.13	86.63	0.50	0.29	0.45
Mucor group	98	60.50	37.42	1.62	8.86	0.88
Penicillium chrysogenum	42	3.13	1.92	1.63	3.24	1.40
Rhizopus stolonifer	50	1.84	1.83	1.00	1.80	0.93

a Presented are percent prevalences, geometric mean concentrations of 35 mold species, GM ratios (MDHs/RHs) for all samples, and GM ratios separately for samples collected during periods of permanent snow cover (January to March) and non-permanent snow cover (April to December).

b >DL, above the detection limit.

By doing so, we created the FERMI metric, which consists of 10 mold species (7 group 1 molds and 3 group 2 molds) (Table 4). The calculation of the FERMI followed the original ERMI approach (see Materials and Methods). In order to keep the majority of FERMI values positive and somewhat numerically comparable with the initial ERMI scale, we adjust here for the mean numerical difference of the FERMI versus ERMI in Finnish LUKAS2 homes (14.42) and add this value to the equation of the FERMI:

(2) $$\text{FERMI}\;=\;\Sigma_{i\;=\;1}^{7}\log_{10}(S_{1i})\;-\;\Sigma_{j\;=\;1}^{3}\log_{10}\left(S_{1j}\right)+14.42$$

**TABLE 4:** The Finnish environmental moldiness index^*a*^

Mold	GM ratio of MDHs/RHs	Prevalence (%) in:	
		MDHs	RHs
FERMI group 1			
Aspergillus ochraceus	2.59	65	31
Aspergillus versicolor	2.08	30	14
Chaetomium globosum	1.50	45	48
Cladosporium sphaerospermum	2.40	90	83
Penicillium corylophilum	1.92	60	45
Penicillium crustosum	1.67	55	48
Penicillium chrysogenum	1.63	45	41
FERMI group 2			
Alternaria alternata	0.90	95	95
Cladosporium cladosporioides type 1	0.55	100	100
Epicoccum nigrum	0.50	100	100

a Shown are data for the mold species/group qPCRs that constitute FERMI groups 1 and 2, their GM ratios, and proportions with detectable levels in homes with severe moisture damage (*n* = 20) versus reference homes without observed moisture damage or mold (*n* = 42).

**FIG 1:**
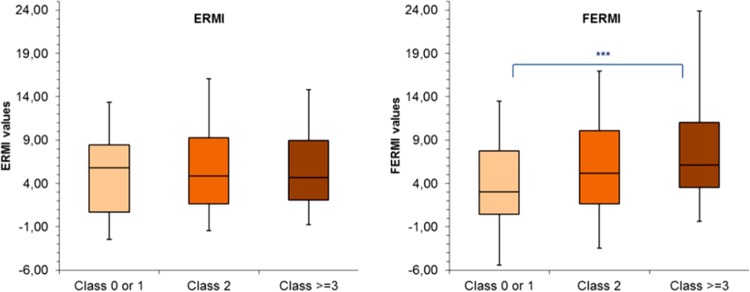
Box plots of ERMI and FERMI values for LUKAS2 homes based on their overall need for repair due to moisture damages. Homes are categorized into need-for-repair class 0 or 1 (no need for repair or only esthetic repairs) (*n* = 60), class 2 (repair of surface materials needed) (*n* = 50), and class 3 or higher (repair of structural components or more extensive repairs needed) (*n* = 34). Boxes represent 25th, 50th, and 75th percentiles; whiskers are 5th and 95th percentiles. ***, *P* value of <0.05 according to Scheffé's pairwise analysis.

When applied to the full sample of 144 homes from the LUKAS2 cohort, the FERMI metric was found to be significantly associated with observations of visible mold in various locations and—this being a clear improvement compared to the original ERMI metric—also with observations of moisture damage in the living room, the child's main living areas, and the whole house (Table 5) as well as with mold odor observed in the whole house (data not shown). Relating to the need for repair in the house, a scale which is based on the severity of moisture problems assessed during the building inspection, the FERMI, unlike the ERMI, showed a significant dose-response association (Fig. 1).

**TABLE 5:** Comparison of mean FERMI values for LUKAS2 homes in which observations of visible mold or more generally moisture damage were made in the living room, the child's main living areas, or the whole house^*a*^

Area in home	Visible mold and FERMI				Moisture damage and FERMI			
	Detection	No. of homes	FERMI^*b*^	*P* value	Detection	No. of homes	FERMI^*b*^	*P* value
Living room	No	141	5.33		None	128	4.98	
	Yes	3	15.20	0.007	Minor	12	11.10	
					Major	4	6.53	0.01
Child's main living area	No	134	4.92		None	102	4.85	
	Yes	10	13.69	<0.0001	Minor	31	6.30	
					Major	11	9.70	0.04
Whole house	No	100	4.51		Class 0/1	60	3.81	
	Yes	44	7.85	0.003	Class 2	50	5.91	
					Class ≥3	34	8.02	0.007

a Differences in the mean FERMI values were evaluated by using a *t* test or one-way ANOVA.

b Mean FERMI value.

The season of dust sampling did not change the significance of associations observed between the FERMI and different moisture damage and dampness indicators in most cases. We did, however, observe significantly higher FERMI and ERMI values for winter than for nonwinter samples (differences in means were 3.3 and 3.4 points, respectively). This finding is likely explained by a great reduction of outdoor mold (group 2) sources during winter (29) but not of indoor sources and moisture damage-related molds (group 1), which results in a higher (F)ERMI value. This observation is relevant for future studies that apply the ERMI or FERMI to sample materials collected during different seasons, especially in countries with distinct seasonal differences in their climates.

The results of our study are encouraging in that the FERMI appears to be a promising tool to confirm inspection-based observations of mold and moisture damage in homes in Finland in an objective way. Our study is limited to a cohort of 144 homes located in Eastern Finland, and the definition of the FERMI was made based on a subsample (*n* = 62) of these homes. Thus, our findings will have to be confirmed in other studies in Finland, before application of the FERMI in research or practical settings can be recommended. Also, the focus of this study was on quantifying moisture damage and mold contamination. The applicability of the FERMI metric to predict respiratory symptoms and the development of asthma due to indoor mold contamination in Finland, similar to what has been done for the ERMI in U.S. homes (12–15), will be the objective of future research efforts.

In conclusion, we show here that remodeling of the ERMI scale to account for local microbial flora and moisture damage characteristics in Finland resulted in a metric with greater potential to objectively rate homes with moisture and mold damage in this specific setting. Following such an approach may also be applicable to other climates, countries, or regions.

## References

[1] Nevalainen A, Partanen P, Jääskeläinen E, Hyvärinen A, Koskinen O, Meklin T, Vahteristo M, Koivisto J, Husman T 1998 Prevalence of moisture problems in Finnish houses. Indoor Air 8:45–49. doi:10.1111/j.1600-0668.1998.tb00007.x.

[2] Karvonen AM, Hyvärinen A, Korppi M, Haverinen-Shaughnessy U, Renz H, Pfefferle PI, Remes S, Genuneit J, Pekkanen J 2015 Moisture damage and asthma: a birth cohort study. Pediatrics 135:e598–e606. doi:10.1542/peds.2014-1239. 25687143

[3] Haverinen-Shaughnessy U, Borras-Santos A, Turunen M, Zock JP, Jacobs J, Krop EJ, Casas L, Shaughnessy R, Täubel M, Heederik D, Hyvärinen A, Pekkanen J, Nevalainen A, HITEA Study Group 2012 Occurrence of moisture problems in schools in three countries from different climatic regions of Europe based on questionnaires and building inspections—the HITEA study. Indoor Air 22:457–466. doi:10.1111/j.1600-0668.2012.00780.x. 22404345

[4] Institute of Medicine, National Academies of Science. 2004 Damp indoor spaces and health. National Academies Press, Washington, DC. 25009878

[5] Pekkanen J, Hyvärinen A, Haverinen-Shaughnessy U, Korppi M, Putus T, Nevalainen A 2007 Moisture damage and childhood asthma: a population-based incident case-control study. Eur Respir J 29:509–515. doi:10.1183/09031936.00040806. 17107993

[6] World Health Organization. 2009 WHO guidelines for indoor air quality: dampness and mould. WHO, Copenhagen, Denmark. 23785740

[7] Mendell MJ, Mirer AG, Cheung K, Tong M, Douwes J 2011 Respiratory and allergic health effects of dampness, mold, and dampness-related agents: a review of the epidemiologic evidence. Environ Health Perspect 119:748–756. doi:10.1289/ehp.1002410. 21269928 PMC3114807

[8] Kanchongkittiphon W, Mendell MJ, Gaffin JM, Wang G, Phipatanakul W 2015 Indoor environmental exposures and exacerbation of asthma: an update to the 2000 review by the Institute of Medicine. Environ Health Perspect 123:6–20. doi:10.1289/ehp.1307922. 25303775 PMC4286274

[9] Vesper S, McKinstry C, Cox D, Dewalt G 2009 Correlation between ERMI values and other moisture and mold assessments of homes in the American Healthy Homes Survey. J Urban Health 86:850–860. doi:10.1007/s11524-009-9384-1. 19536652 PMC2791814

[10] Vesper S, McKinstry C, Haugland R, Wymer L, Bradham K, Ashley P, Cox D, Dewalt G, Friedman W 2007 Development of an environmental relative moldiness index for homes in the U.S. J Occup Environ Med 49:829–833. doi:10.1097/JOM.0b013e3181255e98. 17693779

[11] Vesper SJ, Varma M, Wymer LJ, Dearborn DG, Sobolewski J, Haugland RA 2004 Quantitative polymerase chain reaction analysis of fungi in dust from homes of infants who developed idiopathic pulmonary hemorrhaging. J Occup Environ Med 46:596–601. doi:10.1097/01.jom.0000128160.17144.6e. 15213523

[12] Rosenbaum PF, Crawford JA, Hunt A, Vesper SJ, Abraham JL 2015 Environmental relative moldiness index and associations with home characteristics and infant wheeze. J Occup Environ Hyg 12:29–36. doi:10.1080/15459624.2014.933958. 25068535

[13] Reponen T, Vesper S, Levin L, Johansson E, Ryan P, Burkle J, Grinshpun SA, Zheng S, Bernstein DI, Lockey J, Villareal M, Khurana Hershey GK, LeMasters G 2011 High environmental relative moldiness index during infancy as a predictor of asthma at 7 years of age. Ann Allergy Asthma Immunol 107:120–126. doi:10.1016/j.anai.2011.04.018. 21802019 PMC11610244

[14] Vesper S, McKinstry C, Haugland R, Neas L, Hudgens E, Heidenfelder B, Gallagher J 2008 Higher environmental relative moldiness index (ERMIsm) values measured in Detroit homes of severely asthmatic children. Sci Total Environ 394:192–196. doi:10.1016/j.scitotenv.2008.01.031. 18280542

[15] Blanc PD, Quinlan PJ, Katz PP, Balmes JR, Trupin L, Cisternas MG, Wymer L, Vesper SJ 2013 Higher environmental relative moldiness index values measured in homes of adults with asthma, rhinitis, or both conditions. Environ Res 122:98–101. doi:10.1016/j.envres.2013.01.002. 23419817 PMC3602382

[16] Méheust D, Gangneux JP, Reponen T, Wymer L, Vesper S, Le Cann P 2012 Correlation between environmental relative moldiness index (ERMI) values in French dwellings and other measures of fungal contamination. Sci Total Environ 438:319–324. doi:10.1016/j.scitotenv.2012.08.085. 23022719

[17] Goh V, Yap HM, Gutiérrez RA, Ng LC, Vesper SS 2014 DNA-based analyses of molds in Singapore public buildings results in a proposed Singapore environmental relative moldiness index. Trop Biomed 31:663–669. 25776591

[18] McSharry C, Vesper S, Wymer L, Howieson S, Chaudhuri R, Wright GR, Thomson NC 2015 Decreased FEV1% in asthmatic adults in Scottish homes with high environmental relative moldiness index values. Clin Exp Allergy 45:902–907. doi:10.1111/cea.12482. 25580663 PMC7162076

[19] Karvonen AM, Hyvärinen A, Roponen M, Hoffmann M, Korppi M, Remes S, von Mutius E, Nevalainen A, Pekkanen J 2009 Confirmed moisture damage at home, respiratory symptoms and atopy in early life: a birth-cohort study. Pediatrics 124:e329–e338. doi:10.1542/peds.2008-1590. 19651571

[20] Karvonen AM, Hyvärinen A, Rintala H, Korppi M, Täubel M, Doekes G, Gehring U, Renz H, Pfefferle PI, Genuneit J, Keski-Nisula L, Remes S, Lampi J, von Mutius E, Pekkanen J 2014 Quantity and diversity of environmental microbial exposure and development of asthma: a birth cohort study. Allergy 69:1092–1101. doi:10.1111/all.12439. 24931137 PMC4143956

[21] Haugland RA, Varma M, Wymer LJ, Vesper SJ 2004 Quantitative PCR of selected Aspergillus, Penicillium and Paecilomyces species. Syst Appl Microbiol 27:198–210. doi:10.1078/072320204322881826. 15046309

[22] Haugland RA, Vesper SJ 5 2002 Identification and quantification of specific fungi and bacteria. U.S. patent 6,387,652.

[23] Nevalainen A, Seuri M 2005 Of microbes and men. Indoor Air 15:58–64. doi:10.1111/j.1600-0668.2005.00344.x. 15910530

[24] Amend AS, Seifert KA, Samson R, Bruns TD 2010 Indoor fungal composition is geographically patterned and more diverse in temperate zones than in the tropics. Proc Natl Acad Sci U S A 107:13748–13753. doi:10.1073/pnas.1000454107. 20616017 PMC2922287

[25] Martiny JBH, Bohannan BJ, Brown JH, Colwell RK, Fuhrman JA, Green JL, Horner-Devine MC, Kane M, Krumins JA, Kuske CR, Morin PJ, Naeem S, Ovreås L, Reysenbach AL, Smith VH, Staley JT 2006 Microbial biogeography: putting microorganisms on the map. Nat Rev Microbiol 4:102–112. doi:10.1038/nrmicro1341. 16415926

[26] Barberàn A, Ladau J, Leff JW, Pollard KS, Menninger HL, Dunn RR, Fierer N 2015 Continental-scale distribution of dust-associated bacteria and fungi. Proc Natl Acad Sci U S A 112:5756–5761. doi:10.1073/pnas.1420815112. 25902536 PMC4426398

[27] Tischer C, Zock JP, Valkonen M, Doekes G, Guerra S, Heederik D, Jarvis D, Norbäck D, Olivieri M, Sunyer J, Svanes C, Täubel M, Thiering E, Berlato G, Hyvärinen A, Heinrich J 2015 Predictors of microbial agents in dust and respiratory health in the Ecrhs. BMC Pulm Med 15:48. doi:10.1186/s12890-015-0042-y. 25929252 PMC4425915

[28] Jacobs J, Borràs-Santos A, Krop E, Täubel M, Leppänen H, Haverinen-Shaughnessy U, Pekkanen J, Hyvärinen A, Doekes G, Zock JP, Heederik D 2014 Dampness, bacterial and fungal components in dust in primary schools and respiratory health in schoolchildren across Europe. Occup Environ Med 71:704–712. doi:10.1136/oemed-2014-102246. 25035116

[29] Reponen T, Nevalainen A, Jantunen M, Pellikka M, Kalliokoski P 1992 Normal range criteria for indoor bacteria and fungal spores in a subarctic climate. Indoor Air 2:26–31. doi:10.1111/j.1600-0668.1992.03-21.x.

